# *Agasicles hygrophila* attack increases nerolidol synthase gene expression in *Alternanthera philoxeroides*, facilitating host finding

**DOI:** 10.1038/s41598-020-73130-z

**Published:** 2020-10-12

**Authors:** Yuanxin Wang, Yanhong Liu, Xingchun Wang, Dong Jia, Jun Hu, Ling-Ling Gao, Ruiyan Ma

**Affiliations:** 1 College of Plant Protection, Shanxi Agricultural University, Taigu, 030801 Shanxi People’s Republic of China; 2 College of Life Sciences, Shanxi Agricultural University, Taigu, 030801 Shanxi People’s Republic of China; 3 CSIRO Agriculture and Food, Centre for Environment and Life Sciences, Wembley, WA 6014 Australia

**Keywords:** Herbivory, Wounding, Entomology

## Abstract

Herbivorous insects use plant volatile compounds to find their host plants for feeding and egg deposition. The monophagous beetle *Agasicles hygrophila* uses a volatile (E)-4,8-dimethyl-1,3,7-nonanetriene (DMNT) to recognize its host plant *Alternanthera philoxeroides*. *Alternanthera philoxeroides* releases DMNT in response to *A. hygrophila* attack and nerolidol synthase (NES) is a key enzyme in DMNT biosynthesis; however, the effect of *A. hygrophila* on *NES* expression remains unclear. In this study, the *A. philoxeroides* transcriptome was sequenced and six putative *NES* genes belonging to the terpene synthase-g family were characterized. The expression of these *NES* genes was assayed at different times following *A. hygrophila* contact, feeding or mechanical wounding. Results showed that *A. hygrophila* contact and feeding induced *NES* expression more rapidly and more intensely than mechanical wounding alone. This may account for a large release of DMNT following *A. hygrophila* feeding in a previous study and subsequently facilitate *A. hygrophila* to find host plants. Our research provides a powerful genetic platform for studying invasive plants and lays the foundation for further elucidating the molecular mechanisms of the interaction between *A. philoxeroides* and its specialist *A. hygrophila*.

## Introduction

Alligator weed, *Alternanthera philoxeroides* (Mart.) Griseb. (Caryophyllales: Amaranthaceae), is an invasive weed that has spread across 30 countries, including the USA, New Zealand, and China^1^. It not only causes serious economic losses by hindering river transportation and crop production, but also has a detrimental impact on native wildlife, causing many ecological problems in the invaded areas^2–4^. The most successful method of effectively controlling *A. philoxeroides* is the use of the alligator weed flea beetle *Agasicles hygrophila* (Selman and Vogt) (Coleoptera: Chrysomelidae), which is stably monophagous and has a high food requirement^1^. In China, the safety of using *A. hygrophila* near local crops was evaluated, revealing that the specific diet of this beetle did not change even many years after its introduction^5,6^. This stable and specific feeding relationship means that *A. hygrophila* and its host plant *A. philoxeroides* can be an useful model to study the interaction between plants and their host-specific herbivorous insects; however, few reports have been published on this particular relationship.

Research into the specific interaction between *A. hygrophila* and *A. philoxeroides* has largely focused on surface phenomena, such as the biological performance, life history, and host selection of *A. hygrophila*^2,5,6^. Recently, this plant–insect specificity was further elucidated in a report that host specificity is related to the response of *A. hygrophila* to specialized volatiles released by the plant, which can be detected at a distance^7^. This laboratory-based study found that under undamaged conditions the main volatile of *A. philoxeroides*, homoterpene (*E*)-4,8-dimethyl-1,3,7-nonanetriene (DMNT), was present in larger quantities in this species than in other plants tested. *Agasicles hygrophila* prefers the volatile emitted from plants fed by conspecific insects for 24 h than those from healthy plants, because insect injured plants released more DMNT. DMNT is a significant attractant of *A. hygrophila*, allowing *A. hygrophila* to discriminate host from non-host plants. DMNT is therefore considered to be an important volatile cue for *A. hygrophila* to orient, locate, and feed on *A. philoxeroides*.

DMNT is an important plant terpenoid volatile compound, emitted by the flowers of night-scented plants and plant leaves damaged by herbivores^8,9^, and is thought to attract pollinators or take part in indirect defense to reduce feeding damage and enhance plant fitness^10,11^. Undamaged individuals of most plant species do not release DMNT, or only release a small amount, however, healthy *A. philoxeroides* plants also emit DMNT^7^, which is a derivative of nerolidol. The terpene synthase enzyme nerolidol synthase (NES) was previously reported in lima beans (*Phaseolus lunatus* L.), maize (*Zea mays* L.), and cucumber (*Cucumis sativus* L.), and was shown to catalyze the biosynthesis of the DMNT precursor nerolidol from farnesyl pyrophosphate (FPP)^12–14^. After generating nerolidol, the P450 enzyme takes part in the further cleavage reaction to produce homoterpene DMNT^15^. During this process, terpene synthases determine the type and the quantity of terpenoids produced^16^. Here, nerolidol synthase belongs to the terpene synthase g (TPS-g) subfamily, of which proteins use FPP, geranyl pyrophosphate (GPP) or geranylgeranyl pyrophosphate (GGPP) as substrates to catalyze the production of acyclic monoterpenes^16^.

The expression of terpene synthase genes is involved in the interactions between plants and biotic environmental factors. For example, the linalool produced by transgenic *Arabidopsis thaliana* expressing *FaNES1* from cultivated strawberry (*Fragaria* × *ananassa* Duch.) has a repellent effect on the green peach aphid (*Myzus persicae* (Sulzer)), while the volatiles produced by these plants ((3, S)-(*E*)-nerolidol and DMNT) attract predatory mites such as *Phytoseiulus persimilis* Athias-Henriot^17,18^. Likewise, transgenic chrysanthemum (*Chrysanthemum morifolium* Ramat.) expressing *FaNES1* releases linalool and DMNT, rapidly attracting western flower thrips (*Frankliniella occidentalis* (Pergande))^19^. Transgenic rice (*Oryza sativa* L.) expressing a DMNT biosynthesis gene *Pltps4* from *P. lunatus* enhanced the attraction of *Cotesia chilonis* (Matsumura), a parasitoid of trunk borers^20^. However, there is no report on the expression of *NES* genes in *A. philoxeroides* and the associated effect on *A. hygrophila*.

To elucidate the specific interaction between *A. philoxeroides* and *A. hygrophila*, we explored the role of the *A. philoxeroides NES* genes in this relationship. When we began this study, we lacked available *A. philoxeroides* gene data; therefore, we performed transcriptome sequencing on mixed *A. philoxeroides* tissues and screened the putative *NES* genes. In a previous study, *A. hygrophila* adults were placed on *A. philoxeroides* for 24 h to obtain herbivore-damaged plants^7^. However, in general, the biosynthesis of organic volatiles from damaged plants often takes several minutes to several hours, with the release of terpenoids being typically slower than that of the green leaf volatiles (GLVs)^21–23^. It is possible that this long period of wounding could mask changes in the expression levels of the *NES* genes in these plants. Therefore, in the present study, we examined the behavior of *A. hygrophila* adults on the leaves in the first two hours. We then compared the expression of *NES* genes in leaves subjected to contact, feeding by *A. hygrophila*, and mechanical wounding, revealing that *A. hygrophila* contact upregulates *NES* genes in *A. philoxeroides* before feeding, and *A. hygrophila* feeding significantly increases *NES* gene expression. Chemicals secreted by *A. hygrophila* likely induce the release of large amounts of DMNT from bitten *A. philoxeroides*.

## Materials and methods

### Plant and insect

*Alternanthera philoxeroides* was collected from a naturalized population in Yuhuan County, Zhejiang, China, then grown in the glasshouse of Shanxi Agricultural University, Taigu, Shanxi, China. Shoots from a single individual were separately cultured in pots at a constant temperature of 25 ± 1 °C, with 14 h light and 10 h dark.

*Agasicles hygrophila* was obtained from South China Agricultural University, Guangzhou, China, fed with fresh *A. philoxeroides* leaves, and maintained in an insectary at Shanxi Agricultural University for several generations under controlled conditions (25 ± 1 °C, 80 ± 5% relative humidity, and with a 14-h light: 10-h dark photoperiod).

### Transcriptome sequencing, assembly, and annotation

*Alternanthera philoxeroides* individuals with similar growth were selected for sampling. The fourth and fifth pairs of leaves, the third and fourth internodes from the top, flowers, and roots were harvested and frozen in liquid nitrogen for further processing. Total RNA was isolated from each of the four tissues (leaves, stems, flowers, and roots) using TRIzol (Thermo Fisher Scientific, Waltham, MA, USA) and RNA from each tissue was mixed in equal amounts. The RNA was then submitted to Biomarker Technologies (Beijing, China) for transcriptome sequencing on a HiSeq 2500 system (Illumina, San Diego, CA, USA). The sequencing adaptors, primer sequences, and low-quality data (reads that were< 50 bp and contained more N-bases) were removed from the original data, after which the clean reads were assembled using Trinity (Broad Institute, Cambridge, MA, USA)^24^ to obtain transcripts and unigenes. The unigenes were annotated by using them as a template for BLASTX-searches of multiple public repositories of known proteins, including RefSeq non-redundant proteins (NR), UniProtKB/Swiss-Prot^25^, Gene Ontology (GO; https://geneontology.org/)^26^, Clusters of Orthologous Genes (COG; https://www.ncbi.nlm.nih.gov/COG/)^27^, EuKaryotic Orthologous Groups (KOG), Kyoto Encyclopedia of Genes and Genomes (KEGG; https://www.kegg.jp/kegg/kegg1.html)^28^, Pfam (https://pfam.xfam.org/)^29^, and other databases. The results were parsed to calculate the total number of positive identities per query–subject pair and further filtered using an E-value ≤ 1e−05. The KEGG Orthology results of the unigenes were predicted using KOBAS 2.0 (https://kobas.cbi.pku.edu.cn).

### Identification of terpene synthase genes

The identified nerolidol synthase FaNES1 was used to align to the unigene data from the *A. philoxeroides* transcriptome. The Unigenes in the results annotated as “terpene synthase” or “TPS” were then selected and aligned with the NCBI NR database using an online BLAST search (https://blast.ncbi.nlm.nih.gov/Blast.cgi)^30^. The open reading frames (ORFs) were searched with ORFfinder (https://www.ncbi.nlm.nih.gov/orffinder/) and translated into amino acid sequences. DNAMan 7.0 (Lynnon Biosoft, San Ramon, CA, USA) was used to analyze the sequence identity of the *A. philoxeroides* TPS protein sequences. After removing incomplete sequences shorter than 500 bp and those shorter than 150 aa of translated protein sequences, we used the Maximum Likelihood method and JTT + G + I model to construct a phylogenetic tree of the TPS protein sequences in *A. philoxeroides* together with other plant species (Table S1) by MEGA6.0 (https://www.megasoftware.net/)^31^. The phylogenetic tree was verified using 1000 bootstrap replicates and was drawn by iTOL 5.6.1 (https://itol.embl.de/)^32^.

### Investigation of the short-term feeding behavior in *A. hygrophila* adults

Newly emerged adults of *A. hygrophila* were placed in two glass bottles separately by their genders. The genders of adults were distinguished by individual size and abdominal morphological characteristics. Specifically, the female adult was larger than the male. The female adult has a ventral flat, and two abdominal segments at the end of its abdomen exposed outside the elytra. The abdomen of the male adult is covered with the elytra, and an oblong cavity that contains retractable external genitalia is in the 5th abdominal sternite. Ten newly emerged healthy *A. hygrophila* adults (five males and five females) were taken out from the bottles and starved for 12 h, then placed in a cylindrical box 5 cm in diameter and 4 cm in height. At the beginning of the light period, fresh leaves with stem were placed into the containers. The feeding duration of each beetle was observed and recorded for 2 h, and the area consumed by each adult was evaluated after 1 and 2 h using scanning pixel method. Specifically, we scanned a piece of square paper 10 cm in side length, the same leaf before and after insects feeding with a scanner for grayscale scanning. To calculate the gray part pixels, Photoshop CS6 was used. According to the paper size and the number of pixels, the area size of the insect feeding part was calculated as following: Consumed area = (pixel of leaf − pixel of leaf after consumed)·centimeter/pixel ratio. Centimeter/pixel ratio was calculated by Photoshop CS6.0 using a scanned picture contains a square paper 10 cm in side length. Each experiment was repeated three times.

### Analyses of *NES* expression

#### Plant treatments

Plants with similar levels of growth were selected and cultured in cylindrical glass tubes (3.5 cm diameter, 30 cm height) containing 50 ml water until the roots were grown. One of the fourth pair of leaves was covered with a gauze cage (3 cm diameter, 6 cm height). Six newly emerged *A. hygrophila* adults (three males and three females) were starved for 12 h before they were placed in the cage. Starting from the time adults touched the leaves, a 5-min insect contact treatment was recorded as long as no feeding or excretion occurred. After 1 h of feeding, *A. hygrophila* was removed, and the leaves were collected at 0, 15, 30, 60, 180, 360, and 720 min after insect feeding. For each time point, different individual plants were used. Mechanical wounding was used to distinguish the effect caused by *A. hygrophila* secretion or mechanical damage by *A. hygrophila* feeding. The area and duration of mechanical wounding affect the plants’ volatile release^22^. Therefore, a 6-mm aperture puncher was used to mechanically injure the plant leaf margin for about 5 min until the area was similar to the insect feeding area, and the leaves were collected respectively from different individual plant at 0, 15, 30, 60, 180, 360, and 720 min after the mechanical wounding. At each point, a control was also performed. The leaves were frozen with liquid nitrogen for subsequent RNA extraction. Each treatment has five biological replicates, and each contained three leaves from different plant.

#### Sequence search and primer design

The sequences of the housekeeping and *NES* genes were identified in the transcriptome data based on their similarity with the sequences of these genes in other species stored in the NR protein database. The actin gene *ACT*, the α-tubulin gene *TUA*, the β-tubulin gene *TUB*, and the gene encoding ubiquitin-binding enzyme, *UBC*^33^, were selected as the reference genes. Real-time quantitative PCR (qPCR) primers (Table S2) were designed using Primer3 (https://primer3.ut.ee/) and synthesized by Sangon Biotech (Shanghai, China). To check the specificity of all primers, total RNA was reverse transcribed using the PrimeScript RT reagent kit with gDNA eraser (Takara Bio, Kusatsu, Japan). PCR was performed on the cDNA, and the PCR products were analyzed on a 1% agarose gel.

#### qPCR and data analysis

The total RNAs of the collected materials were extracted using the TRIzol method outlined above. The RNAs were quantified using a BioPhotometer Plus (Eppendorf, Hamburg, Germany), and their quality was verified using electrophoresis on an agarose gel. A 1-μg aliquot of total RNA was reverse transcribed at 37 °C for 15 min with an oligo-dT primer using the PrimeScript RT reagent kit with gDNA eraser (Takara Bio, Kusatsu, Japan). The gDNA eraser was used to remove genomic DNA. Two independent reactions were performed for each RNA sample.

The qPCR was performed using an Applied Biosystems 7500 Fast Real-Time Detection system (Thermo Fisher Scientific, Waltham, MA, USA) with the SYBR Green Realtime PCR Master Mix (Toyobo, Osaka, Japan). Each reaction was carried out according to the manufacturer’s protocol in a 20-µl volume, which contained 2 µl tenfold diluted reverse-transcribed cDNA as a template and 500 nM of each primer. In all qPCR experiments, the transcript levels of three independent biological replicates were measured, with three technical replicates performed for each sample. A no-template control was included in each run for each gene. The qPCR reaction was performed using the following conditions: 95 °C for 2 min, followed by 40 cycles of 95 °C for 15 s, 55 °C for 15 s, and 72 °C for 30 s. Finally, a melting curve was generated by increasing the temperature from 65 °C to 95 °C to determine the specificity of the reactions.

The stability of the four candidate reference genes *UBC*, *ACT*, *TUA*, and *TUB* were evaluated in the control group, *A. hygrophila* contact group, *A. hygrophila* feeding group, and mechanical wounding group using the reference screening software geNorm^34^, NormFinder^35^, and BestKeeper^36^. The most stable reference gene, *UBC*, was selected as the reference for the qPCR experiment.

The normalized expression values of the genes of interest were calculated using the 2^–∆∆*C*t^ method^37^. For calculating the basal gene expression in the control group, *ApTPS19* was used as the control gene and the basal expression of *ApTPS19* was normalized equal to 1. Other *NES* gene basal expression levels in the *A. philoxeroides* control group leaves were calculated and compared as the folds of *ApTPS19* basal expression. The transcript levels of the *NES* genes in the control leaves and each of those subjected to contact, feeding by *A. hygrophila*, and mechanical wounding were transformed with logarithmic transformation to ensure normal distribution and compared using independent two-sample Student’s *t*-tests performed in IBM SPSS Statistics 22.0 (International Business Machines Corporation, Amonk, New York, USA). One-way ANOVA was used to analyze the basal expression of each *NES* gene. Two-way ANOVA was used to analyze the expression of each *NES* gene in each treatment group, with treatment methods and post-treatment time as the influential factors. Two-way ANOVAs were also used to analyze the expression of the *NES* genes in the four treatment groups at the same time point. Multiple comparisons were performed using Tukey’s post-hoc test. Student’s *t*-test was used to compare the expression levels of *NES* genes between two different treatments, after the homogeneity test.

## Results

### RNA sequencing, assembly, and annotation

To identify the terpene synthase genes involved in DMNT biosynthesis in *A. philoxeroides*, we performed transcriptome sequencing on the major tissues of this plant. A total of 9.91 Gb nucleotides and 40,984,113 raw reads were obtained from the *A. philoxeroides* sequencing, and the percentage of Q30 bases reached 91.14%. The original data were deposited in the NCBI SRA library under the accession number SRR10537333. In total, 95.97% of the clean reads were assembled into 4,904,951 contigs, 182,318 transcripts, and 83,179 unigenes (Table 1).

**Table 1:** Summary of the RNA-seq data and the de novo assembly of the *A. philoxeroides* unigenes.

Sequencing	
Raw reads	40,984,113
Clean reads	39,332,453
Q30	91.14%
N	0.01%
N50	1103
Unigene number	83,179
Unigene length (nt)	67,987,255
Mean length (nt)	817.36
Unigenes annotations based on homology with the NR protein database	63,076
Unigenes annotations based on homology with the KEGG database	23,984
Unigenes annotations based on homology with the COG database	20,587
Unigenes annotations based on homology with the GO database	42,386

**Figure 1:**
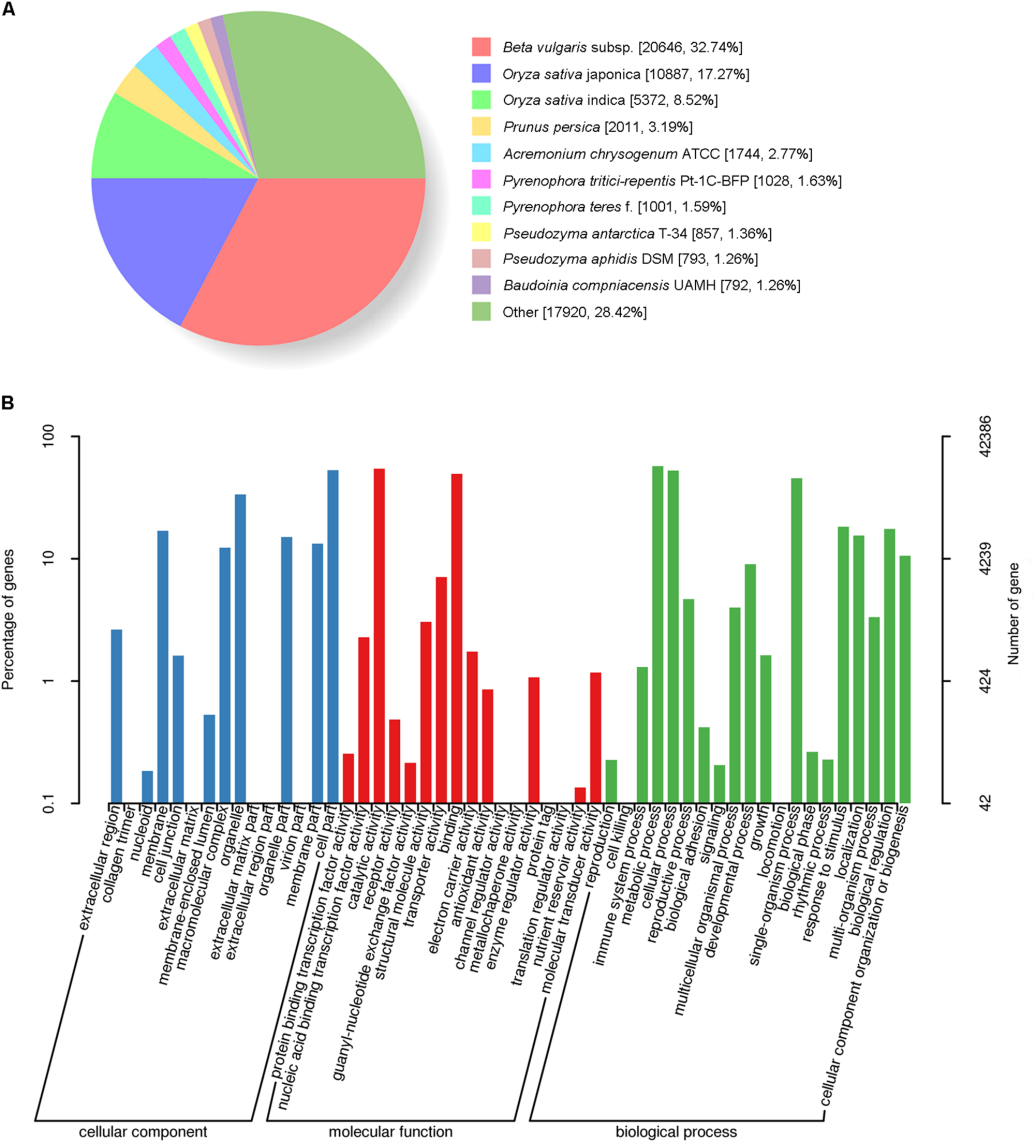
Species with high levels of similarity in the NR database and GO classification statistics of *A. philoxeroides* annotated unigenes. (**A**) Species with relatively high levels of similarity to the *A. philoxeroides* unigenes in the NR protein database; (**B**) GO classification statistics of *A. philoxeroides* annotated unigenes.

Homologs of the unigenes were identified by BLASTX; each publicly available protein database was searched using the transcriptome data, which resulted in the successful annotation of 63,536 (76.4%) of the unigenes. Among them, 63,076 genes were annotated based on their homology with sequences in the NR protein database, which accounted for 99.28% of the total number of annotations (Table 1). 32.74% of *A. philoxeroides* annotated sequences were aligned to beet (*Beta vulgaris* subsp. *vulgaris* L.) (Fig. 1A).

We classified the predicted genes of *A. philoxeroides* using the GO, COG, KOG, KEGG, and other protein databases. A total of 42,386 unigenes were divided into three major categories and 52 functional groups (Fig. 1B); 23,984 unigenes were mapped to 277 KEGG pathways, including the glycolytic, gluconeogenesis, and pentose phosphate pathways, among which the numbers of unigenes associated with the carbon cycle (ko01200), amino acid synthesis (ko01230), and ribosome (ko03010) pathways were 1172, 1057, and 1016, respectively (Table 1; Fig. S1, S2).

### Identification and analysis of the terpene synthase genes and *NES* genes in *A. philoxeroides*

In the *A. philoxeroides* transcriptome data, a total of 34 terpene synthase genes were identified (Table S3). Their encoded protein sequences were compared with those of other species. The sequences of terpene synthase genes in *A. philoxeroides* had the highest similarity to those of *B. vulgaris*.

**Figure 2:**
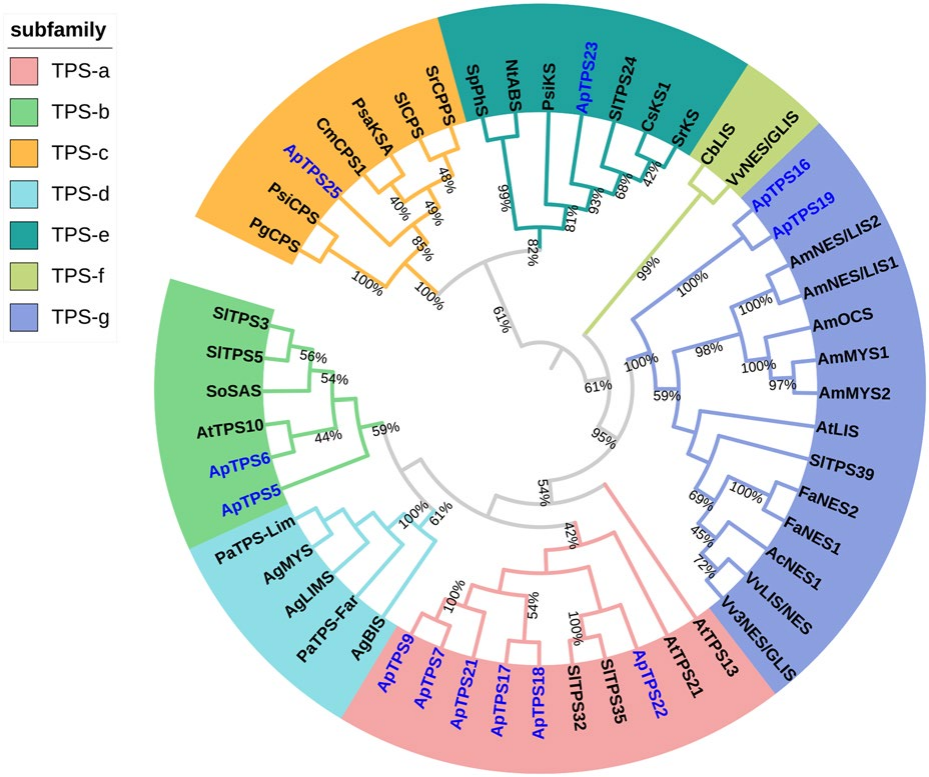
Phylogenetic tree of *A. philoxeroides* terpene synthase (TPSs) together with previously characterized plant TPSs.The tree was created in MEGA6 using the Maximum Likelihood method with JTT + G + I model, verified by bootstrap method, and drawn using iTOL (https://itol.embl.de/). The outermost circle layer indicates the species name and associated clade of each protein. Proteins labeled in blue font are terpene synthase proteins obtained from the *A. philoxeroides* transcriptome. The proteins and accession numbers used in the figure are shown in Table S3. The number on the branches indicate the supporting bootstrap value of proteins on the branches (between 40 and 100%). The closer the bootstrap value is to 1, the higher confident the branch is. The proteins on the same branch mean they are the most related proteins, which may share high homology and have similar function.

The phylogenetic tree showed that the 12 terpene synthase proteins were divided into five subfamilies: TPS-a, TPS-b, TPS-c, TPS-e, and TPS-g (Fig. 2). The TPS-a, TPS-b, and TPS-g subfamilies contained the terpene synthases specific to angiosperm species, which were isolated from the other TPS subfamilies. These angiosperm-specific proteins were class-III terpene synthases, which was in accordance with the species classification of *A. philoxeroides*. ApTPS7, ApTPS9, ApTPS17, ApTPS18, ApTPS21, and ApTPS22 were classified into the TPS-a subfamily; ApTPS5 and ApTPS6 were classified into the TPS-b subfamily; and ApTPS16 and ApTPS19 were classified into TPS-g family. The TPS-e subfamily largely comprised kaurene synthase, which had a function similar to the annotated function of ApTPS23. The TPS-c subfamily mainly contained the monofunctional diterpene synthase, copalyl diphosphate synthase (CPS). ApTPS25, which was annotated as CPS, was also grouped into the TPS-c subfamily.

In total, six *NES* genes were obtained: *ApTPS10*, *ApTPS12*, *ApTPS14*, *ApTPS15*, *ApTPS16*, and *ApTPS19* (Table 2). *ApTPS19* was the longest of these genes (2161 bp), and was predicted to have an ORF of 1791 bp and to encode a protein of 596 aa in length. Four NES proteins were removed due to their shorter sequences, leaving ApTPS16 and ApTPS19, both of which were classified into the TPS-g subfamily. These proteins were annotated as NES, and shared about a 40% similarity with the other TPS enzymes in *A. philoxeroides*.

**Table 2:** Nerolidol synthase genes in the *A. philoxeroides* transcriptome.

Genes	Length (bp)	Identity (%)	e-value	Annotated function	Gene accession
*ApTPS10*	656	43	3e^−10^	(3S)-linalool/(E)-nerolidol /(E,E)-geranyl linalool synthase [*Vitis vinifera*]	NP_001267990.1
*ApTPS12*	415	42	3e^–28^	nerolidol synthase [*Tripterygium wilfordii*]	AQA26342.1
*ApTPS14*	336	50	7e^–15^	(3S,6E)-nerolidol synthase 1 [*Beta vulgaris* subsp. *vulgaris*]	XP_010686920.1
*ApTPS15*	830	61	3e^–81^	terpene synthase 3 [*Populus trichocarpa*]	AEI52903.1
*ApTPS16*	1495	46	1e^–123^	(3S)-linalool/(E)-nerolidol /(E,E)-geranyl linalool synthase [*Vitis vinifera*]	ADR74213.1
*ApTPS19*	2161	45	3e^–147^	(3S)-linalool/(E)-nerolidol /(E,E)-geranyl linalool synthase [*Vitis vinifera*]	ADR74213.1

### Short-term feeding behavior of *A. hygrophila*

In the present study, we examined the behavior of *A. hygrophila* adults on the leaves of *A. philoxeroides* within the first two hours. These actions include contacting, eating and resting. The time between the male and female adults touching the leaves and the initiation of feeding was 5.9 min and 6.2 min, respectively, after a 12-h starvation. The duration of a single feed was 19.6 min for males and 26.5 min for females. No statistical differences were observed between the feeding behaviors of male and female adults (Table 3). The interval between the first and second feeding periods varied from 1 to 90 min, during which the adults usually remained inactive. We, therefore, selected a 5-min period after placing the adults into the cage for use as the contact treatment in future experiments.

**Table 3:** Early feeding behaviors of *A. hygrophila* adults.

Gender	Time between touch and feeding (min)	Duration of single feeding behavior (min)	Leaf area consumed by a single adult within 1 h (mm^2^)
Female	6.2 ± 1.27	26.5 ± 2.63	618.91 ± 59.67*
Male	5.9 ± 1.35	19.6 ± 3.17	433.01 ± 28.44

The data in the table are presented as means ± SE. Asterisks (*) indicate a statistical difference between male and female adults, determined using a Student's *t* test (*P* < 0.05).

In addition, the leaf areas consumed by one adult in 1 h and the areas consumed by different numbers of adults in 1 h were also evaluated (Tables 3, S4). The feeding of male and female adults resulted in different leaf areas consumed; therefore, the use of six adults (three males and three females) was chosen as the insect feeding group for the subsequent 1-h feeding experiment. The leaf area consumed was about 20% of the entire leaf using this treatment method, which provided a basis for the mechanical wounding treatment of the *A. philoxeroides* leaves.

### Selection of reference genes in leaves under *A. hydrophila* contact, feeding, or mechanical wounding

To ensure the accuracy of the quantitative analysis of *NES* expression under the different treatment conditions, the expression stabilities of the commonly used reference genes *ACT*, *TUA*, *TUB*, and *UBC* were screened. The results showed that the *C*t values of the four candidate reference genes were 19–26 (Fig. S3), with the lowest *C*t values observed for *UBC* (19–22) and the highest *C*t values observed for *TUB* (23–26). The expression levels of each candidate reference gene were different when they were analyzed using GeNorm, NormFinder, and BestKeeper.

The GeNorm and NormFinder software analysis revealed that the M values of the four reference genes were all less than 1.5, and therefore these genes were considered to be relatively stable when expressed. Among them, *UBC* had the smallest M value and the best expression stability, whereas *TUA* had the largest M value and a relatively poorer stability. The BestKeeper software analysis revealed that the SD values of the *C*t values for the four reference genes were < 1, indicating relatively stable gene expressions. Among them, *UBC* had the smallest SD value and the best expression stability, whereas *TUA* had the largest SD value and the worst stability (Table 4). Based on these results, the expression stabilities of the four candidate reference genes were ranked as follows: *UBC* > *TUB* > *ACT* > *TUA*. We therefore used *UBC* as the reference gene to investigate the expression of the *NES* genes in the leaves of *A. philoxeroides* under the different damage treatments.

**Table 4:** Stable values of data analyses performed using GeNorm, NormFinder, and BestKeeper.

GeNorm		NormFinder		BestKeeper		
Gene name	M	Gene name	M	Gene name	SD	CV
*UBC*	0.776	*UBC*	0.319	*UBC*	0.53	2.60
*TUB*	0.797	*TUB*	0.319	*TUB*	0.59	2.38
*ACT*	0.843	*ACT*	0.431	*ACT*	0.85	3.92
*TUA*	0.968	*TUA*	0.573	*TUA*	0.86	3.67

M dictates the stability index: the larger the M value, the worse the expression stability of the gene. SD is standard deviation of the Ct value. CV is coefficient of variation of the *C*t value. If SD < 1, the gene was considered to be relatively stable; therefore, the smaller the standard deviation and coefficient of variation, the more stable the gene expression.

### Basal expression of various *NES* genes in healthy leaves

**Figure 3:**
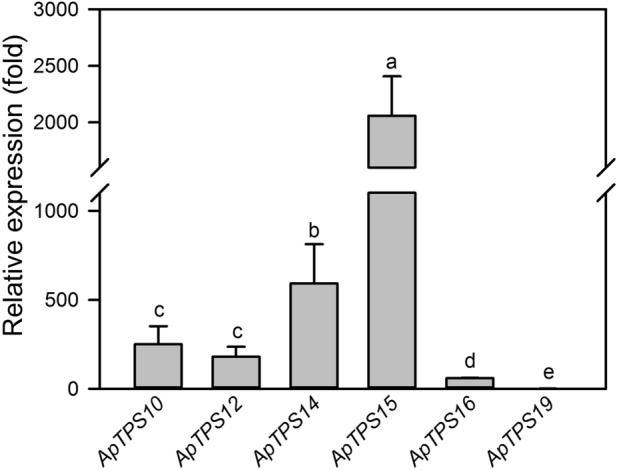
Relative transcription levels of the six nerolidol synthase genes in the plant of *A. philoxeroides* under undamaged control conditions. *UBC* was used as the reference gene to calculate the gene transcript level. The relative expression level of each *NES* is shown in fold relative to that of *ApTPS19* which had the lowest transcription level that was taken as “1”. The values are means ± SD of three biological replications. Different letters indicate statistical differences between the genes, revealed using a one-way ANOVA and a Tukey’s post-hoc test (*P* < 0.05).

We investigated the basal expression level of the *NES* genes in the healthy leaves because of the large amount of DMNT from healthy *A. philoxeroides* plants*.* The results revealed statistical differences in the basal expression of the six *NES* genes in the leaves of the control plants (*P* < 0.05). Among them, *ApTPS19* had the lowest basal expression, while *ApTPS15* had the highest basal expression, which was 2058.9 times higher than that of *ApTPS19* (Fig. 3).

### Induction of *NES* genes in *A. philoxeroides *prior to feeding by *A. hygrophila*

**Figure 4:**
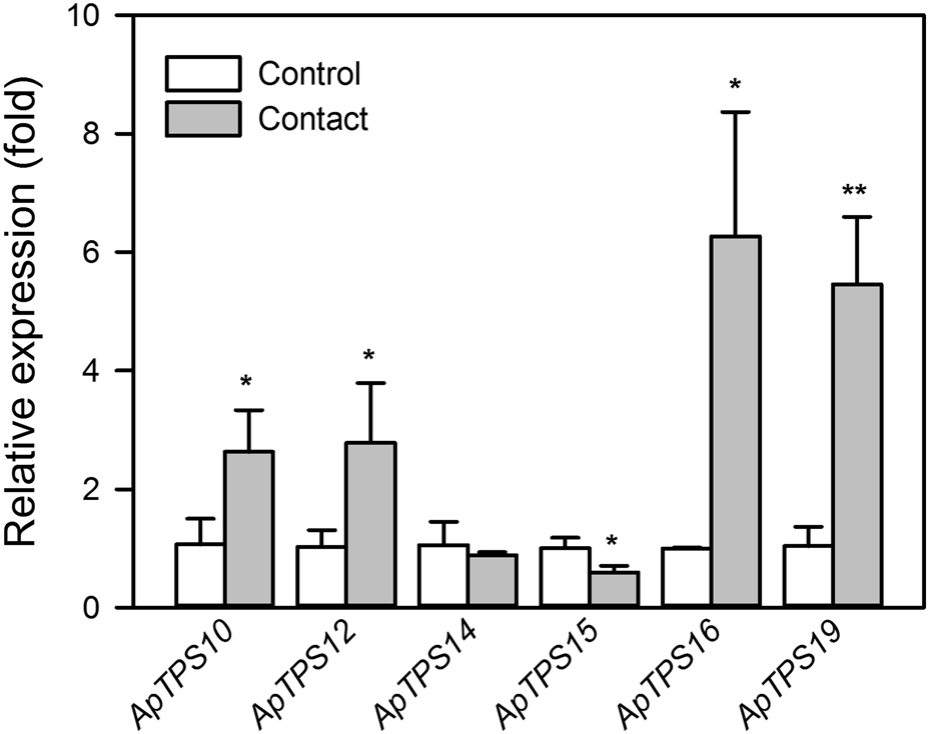
Nerolidol synthase gene expression levels in the control group and the *A. hygrophila* contact group of *A. philoxeroides* plants. *UBC* was selected as the reference gene. The values are means ± SD (normalized that the expression level in the control group equal to 1) of three biological replications. Asterisks indicate statistical differences between the treatments (*P < 0.05; **P < 0.01; Student’s *t*-test).

The early feeding behavior of *A. hygrophila* adults was investigated, revealing that the time between the flea beetles touching the leaves and the initiation of feeding was typically longer than 5 min. The changes in *NES* gene expression were evaluated in the leaves touched by *A. hygrophila* for 5 min. The expression levels of *ApTPS10*, *ApTPS12*, *ApTPS16*, and *ApTPS19* increased significantly in *A. philoxeroides* leaves touched by *A. hygrophila* (*P* < 0.05), with the expression of *ApTPS16* being 6.27 times higher than that of the control group. By contrast, *ApTPS15* expression decreased significantly in the leaves touched by *A. hygrophila* (*P* < 0.05), whereas *ApTPS14* expression was not statistically different between the control and *A. hygrophila* contact group (Fig. 4).

### *A. hygrophila* feeding strongly induces *NES* gene expression in *A. philoxeroides*

**Figure 5:**
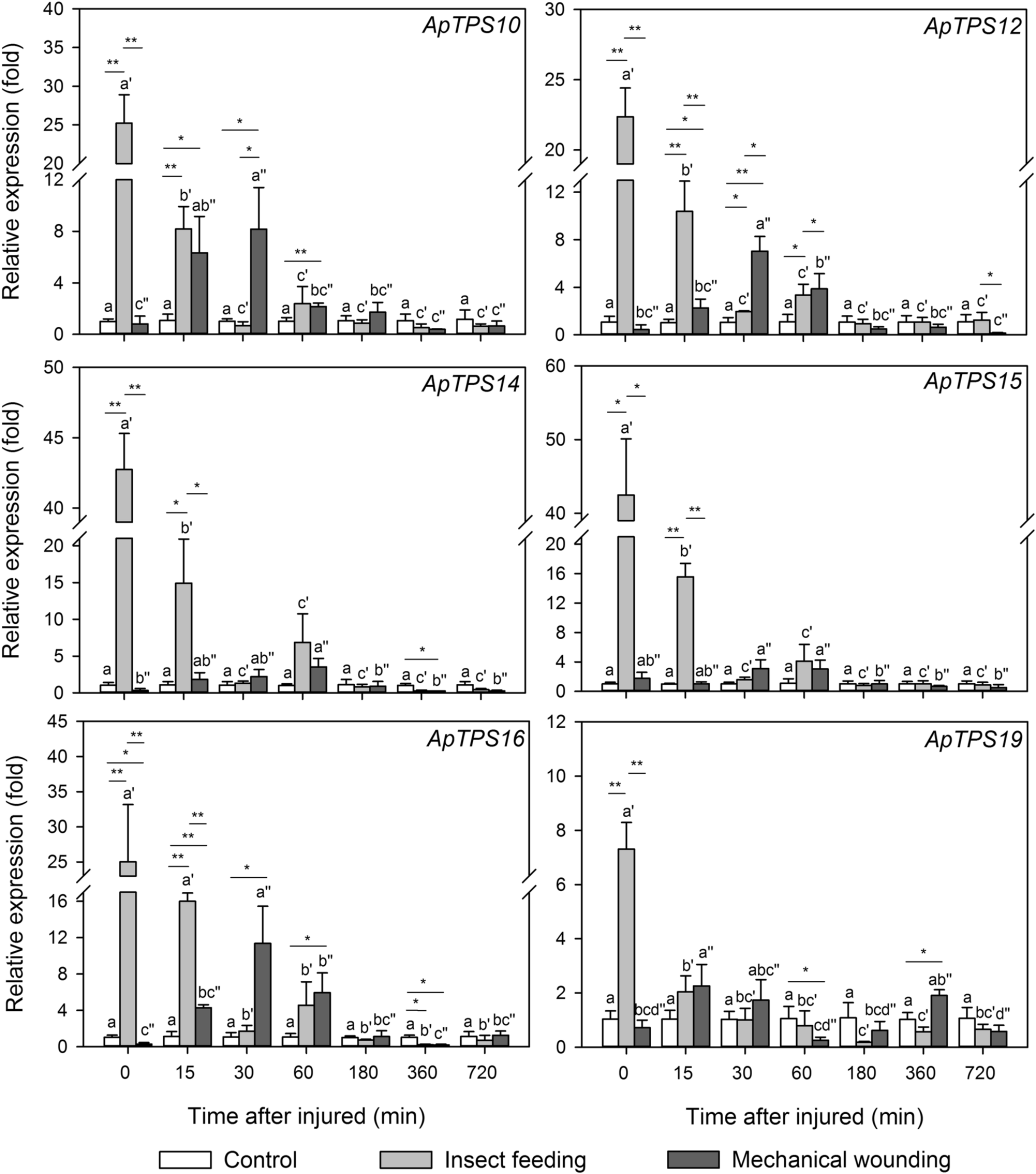
Nerolidol synthase gene expression levels in the control group, *A. hygrophila* feeding group, mechanical wounding group of *A. philoxeroides* plants. In the *A. hygrophila* feeding group, the *A. philoxeroides* leaves were sampled at the indicated time points following a 1-h feeding experiment. In the mechanical wounding group, the *A. philoxeroides* leaves were sampled at the indicated time points following their mechanical damage. The values are means ± SD (normalized that the expression level in the control group equal to 1) of three biological replications. Different letters indicate statistical differences between different time treatments in control group, as determined using a one-way ANOVA and a Tukey’s post-hoc test (*P* < 0.05). Different letters with single quotation mark indicated statistical differences between different time treatments after insect feeding, as determined using a one-way ANOVA and a Tukey’s post-hoc test (*P* < 0.05). Different letters with double quotation marks indicate statistical differences between different time treatments after mechanical wounding, as determined using a one-way ANOVA and a Tukey’s post-hoc test (*P* < 0.05). Asterisks indicate statistical differences between every two treatments in the same time treatment (*P < 0.05; **P < 0.01; Student’s *t*-test).

The changes in the *NES* gene expression upon *A. hygrophila* feeding were also analyzed in a time series. All of the six *NES* genes in the leaves were significantly up-regulated immediately after 1 h insect-damaged (*P* < 0.05) (Fig. 5).

*ApTPS14* expression showed the greatest up-regulation, reaching a level 42.7 times higher than that of the control plants (*P* < 0.05). The expression of *ApTPS19* showed the smallest up-regulation by insect feeding, reaching a level only 7.3 times higher than that of the control plants (*P* < 0.05). The four other *NES* genes showed several-fold up-regulation of their expression following *A. hygrophila* feeding, and were significantly different from the control plants (*P* < 0.05). Fifteen minutes after feeding ended, the expression of the six *NES* genes remained statistically higher than in the control plants (*P* < 0.05), but as time progressed their expression levels gradually decreased, returning to their normal levels within 15 to 30 min. *ApTPS12*, *ApTPS14*, *ApTPS15*, and *ApTPS16* also showed a small peak in expression 1 h after feeding ended (*P* < 0.05) (Fig. 5).

### Mechanical wounding induces delayed and low levels of *NES* gene expression

Upon mechanical wounding, all of six *NES* gene expression showed no statistical difference from that in the leaves of the control group (Fig. 5).

After the mechanical wounding, the expression levels of the differentially expressed *NES* genes first increased to a peak at around 30–60 min, then gradually decreased to a level similar to that of the control plants after 1–3 h. After 30 min, *ApTPS10*, *ApTPS12*, *ApTPS15*, and *ApTPS16* were significantly differentially expressed in the leaves subjected to mechanical wounding in comparison with the control plants (*P* < 0.05). After 1 h, *ApTPS14* expression remained significantly different in the two groups (*P* < 0.05). However, at several time points, *ApTPS19* expression was similar between the two groups (Fig. 5).

## Discussion

Here, we showed that the presence of *A. hygrophila* affects *NES* gene expression in *A. philoxeroides* even before feeding and short-term feeding (1 h), and by *A. hygrophila* strongly and rapidly induces the up-regulation of the *NES* genes. This up-regulation of *NES* expression was likely the main reason why chewed leaves released more DMNT, in turn attracting more *A. hygrophila* to feed on these plants.

Initially, second-generation transcriptome sequencing was performed on the roots, stems, leaves, and flowers of *A. philoxeroides* to provide genetic and protein data that were lacking for this species. Compared with the previously reported transcriptomes of *A. philoxeroides* roots^38^ and *A. philoxeroides* individuals growing in ponds and uplands^33^, our sequencing provided novel genetic data for the flowers, which are an important organ for the release of volatile compounds^39,40^. Also, the annotation of the *A. philoxeroides* genes and proteins was improved. Along with the full-length transcriptome sequencing of *A. hygrophila*^41^, we provided valuable data for the comprehensive study of the molecular mechanisms underlying the specificity between *A. philoxeroides* and *A. hygrophila*. Few studies have reported sequencing data for other species in the *Alternanthera* genus; the genetic and protein data obtained from sequencing other related species in this family are limited to several edible and medicinal crops, such as *Amaranthus caudatus* L.^42^, *Amaranthus hypochondriacus* L.^43–45^, and *Achyranthes bidentata* Blume^46^. The transcriptome data of *A. philoxeroides* has therefore enriched the genetic data available for the Amaranthaceae, especially the *Alternanthera* genus. It also enriched the genetic data available for invasive plants, providing powerful genetic support for exploring their invasive properties, and their interactions with herbivorous insects.

In total, 34 *TPS* genes were predicted in the *A. philoxeroides* transcriptome (Table S3). In plants with published genomes, the number of *TPS* genes ranges from 19 to 152^16^; for example, there are 40 *TPS* genes in *Arabidopsis thaliana*, of which 32 have known or predicted functions^47^. Based on the materials and methods used for this transcriptome sequencing, we speculate that the *A. philoxeroides* genome may contain more *TPS* genes, which should be further explored using genome sequencing. The discovery of these *TPS* genes will enhance our understanding of the *TPS* genes in the Amaranthaceae as a whole.

Feeding by the specialist insect *A. hygrophila* affects *A. philoxeroides*, causing the release of DMNT^7^. The *NES* genes, which belong to TPS-g subfamily, are related to DMNT biosynthesis. Six *NES* genes were screened in this transcriptome. Following the contact or feeding *by A. hygrophila*, the expression pattern of *A. philoxeroides NES* genes was further studied. The *NES* genes were up-regulated before feeding by *A. hygrophila*. In the 5 min before feeding, when adults merely touched the leaves of *A. philoxeroides*, the initial contact caused significant up-regulation of most of the *NES* genes. After injury, plants usually activated a transcription cascade, leading to the expression of genes involved in the release of volatile organic compounds; however, it generally takes a few hours to a day (24 h) for the compounds to be released^48–52^. Compared with the *Lilium* ‘Siberia’ terpene synthase gene *LoTPS1*, which is activated in response to wounding, *NES* genes in *A. philoxeroides* responded to damage much faster^48^.

The rapid up-regulation of these genes following contact with *A. hygrophila* may have two explanations. In the first case, when the beetle touches the leaf surface, it might cause slight mechanical damage to the leaf, which in turn up-regulates *NES* expression. The tarsus of an insect typically has claws, which can cause fine scratches on leaf blades and break their trichomes as the insect crawls along the leaf surface. This has been confirmed in tobacco (*Nicotiana tabacum* L.), soybean (*Glycine max* (L.) Merr.)^53,54^, and tomato (*Solanum lycopersicum* L.)^55^. Also, the minor mechanical damage causes the release of plant terpenoid volatiles, which has been documented in a touch study of potato (*Solanum tuberosum* L.)^56^. However, the expression level of *NES* in the *A. hygrophila* contact group was significantly higher than that in the mechanical wounding group. The other possibility is the chemical substances secreted by insects may generate a chemical “footprint” on the leaf and up-regulate the expression of the *NES* genes. In other species, insect chemical footprints have been shown to mediate the transmission of the insect’s intraspecific and interspecific information. For example, some pollinators has been showed to transmit flower information to the same species by chemical “footprint”^57,58^, some parasitoids have located host insects by recognition of the chemical traces that host insects deposited after crawling^59^. It is not yet clear whether these secretions can trigger plant responses^60^, a hypothesis that will need to be tested in future studies.

When *A. hygrophila* was in contact with the plant for prolonged periods of time, the beetles began to feed. Short-term feeding (1 h) by *A. hygrophila* caused rapid and intense up-regulation of *NES* genes, which is consistent with the previous study showing release of large amounts of DMNT after feeding by *A. hygrophila*^7^. Plants responded quickly to insects’ damage, including the rapid biosynthesis of monoterpenes and sesquiterpenes^61^. For example, the feeding behavior of insect herbivores such as *Spodoptera littoralis* (Boisduval)^62^, *Lymantria dispar* L.^63^, and *Hylobius abietis* (L.)^64^ also induced the expression of plant terpene synthase genes and the release of terpene volatiles. For insects with chewing mouthparts, such as *A. hygrophila*, the feeding behavior not only causes mechanical wounding to the leaves but also deposes oral secretions, such as saliva, in the leaf incisions. Both of these can induce plant volatile production through special transcriptional pathways^65^. In this study, we used a puncher simulating similar feeding areas to rule out the effect of mechanical wounding on gene expression changes. However, *NES* expression in the *A. hygrophila* feeding group was still significantly greater than that in the mechanical wounding group. In our previously work, we measured the DMNT released from *A. philoxeroides* following mechanical wounding and feeding by *A. hygrophila*. The DMNT released due to insect feeding was 240 units ng·h^−1^·10 g^−1^·FW^−1^; by contrast, the DMNT release by mechanical wounding was only 13 units ng·h^−1^·10 g^−1^·FW^−17^. The magnitudes of *NES* expression caused by insect feeding and mechanical wounding in this study reflected the magnitudes of DMNT in response to these two treatments, respectively^7^. In Li^7^, the DMNT was collected over a 12 h period which covered the time frame used in this study. If there would be any DMNT released by mechanical wounding, it would be very minimal compared to the large quantity of DMNT caused by insect feeding. This suggests that the chemicals secreted by *A. hygrophila* might trigger the up-regulation of *NES*, which leads to the release of a large amount of DMNT after *A. hygrophila* feeding^7^.

In damaged *A. philoxeroide*s leaves, high expression of *NES* genes causes a large amount of DMNT to be released, which attracts more specialist *A. hygrophila*^7^. The release of plant volatiles has a significant attraction to insects (herbivore and their parasitoids) and may provide signals for conspecifics to find host plants or increase aggregation. For example, the root-feeding larvae of a specialized insect *Bikasha collaris* (Baly) can induce its host plant *Triadica sebifera* (L.) Small to produce volatiles, attracting aboveground conspecific adults^66^. Also, *Spodoptera littoralis* caterpillars could be attracted by volatiles emitted by maize (*Zea mays*) damaged by conspecific larvae, and thereby find a less suitable but easily detectable host plant^67^. We speculated that the specialist *A. hygrophila* might use the chemicals it secretes to affect the release of volatiles from the host plants and facilitate population development by making it easier for conspecifics to locate their host plants *A. phyloxeroides*. These questions will need to be explored in future research.

## Conclusions

In this study, we identified six *NES* genes from the *A. philoxeroides* transcriptome. When the leaves were contact by *A. hygrophila*, *NES* gene expression increased rapidly, and was strongly up-regulated after *A. hygrophila* feeding. Our results suggested that the rapid and strong increase of *NES* transcript levels promotes the large-scale release of DMNT. The released DMNT helps conspecific species of *A. hygrophila* locate host plants. Our results also suggested that chemicals secreted by *A. hygrophila* could be responsible for the rapid and strong increase of *NES* transcript levels. Our results represent an initial explanation of the molecular mechanisms underlying the interaction between *A. philoxeroides* and its natural enemy *A. hygrophila* and provide a molecular basis for understanding the relationships between obligate herbivorous insects and their host plants.

## Supplementary information

Supplementary information 1.

Supplementary information 2.

Supplementary information 3.

Supplementary information 4.
