# Supplementary material for: Agasicles hygrophila attack increases nerolidol synthase gene expression in Alternanthera philoxeroides, facilitating host finding
Source: Sci Rep. 2020 Oct 12;10:16994. doi: 10.1038/s41598-020-73130-z (PMC7552398; doi:10.1038/s41598-020-73130-z)
Supplement: Supplementary file 1 — Supplementary information 1. [file 41598_2020_73130_MOESM1_ESM.docx]

***Agasicles hygrophila* attack increases nerolidol synthase gene expression in *Alternanthera philoxeroides*, facilitating host finding**

Yuanxin Wang^1^, Yanhong Liu^1^, Xingchun Wang^2^, Dong Jia^1^, Jun Hu^2^, Ling-Ling Gao^3^*, Ruiyan Ma^1^*

^1^ College of Plant Protection, Shanxi Agricultural University, Taigu, Shanxi, People’s Republic of China

^2^ College of Life sciences, Shanxi Agricultural University, Taigu, Shanxi, People’s Republic of China

^3^ CSIRO Agriculture & Food, Centre for Environment and Life Sciences, Wembley, Western Australia 6014, Australia

*Corresponding authors:

Ruiyan Ma^1^

College of Plant Protection, Shanxi Agricultural University, Taigu 030801, People’s Republic of China

Email address: [maruiyan2019@163.com](mailto:maruiyan2019@163.com)

Ling-Ling Gao^3^

CSIRO Agriculture and Food, Centre for Environment and Life Sciences, Wembley, Western Australia 6014, Australia.

Email address: Lingling.Gao@csiro.au


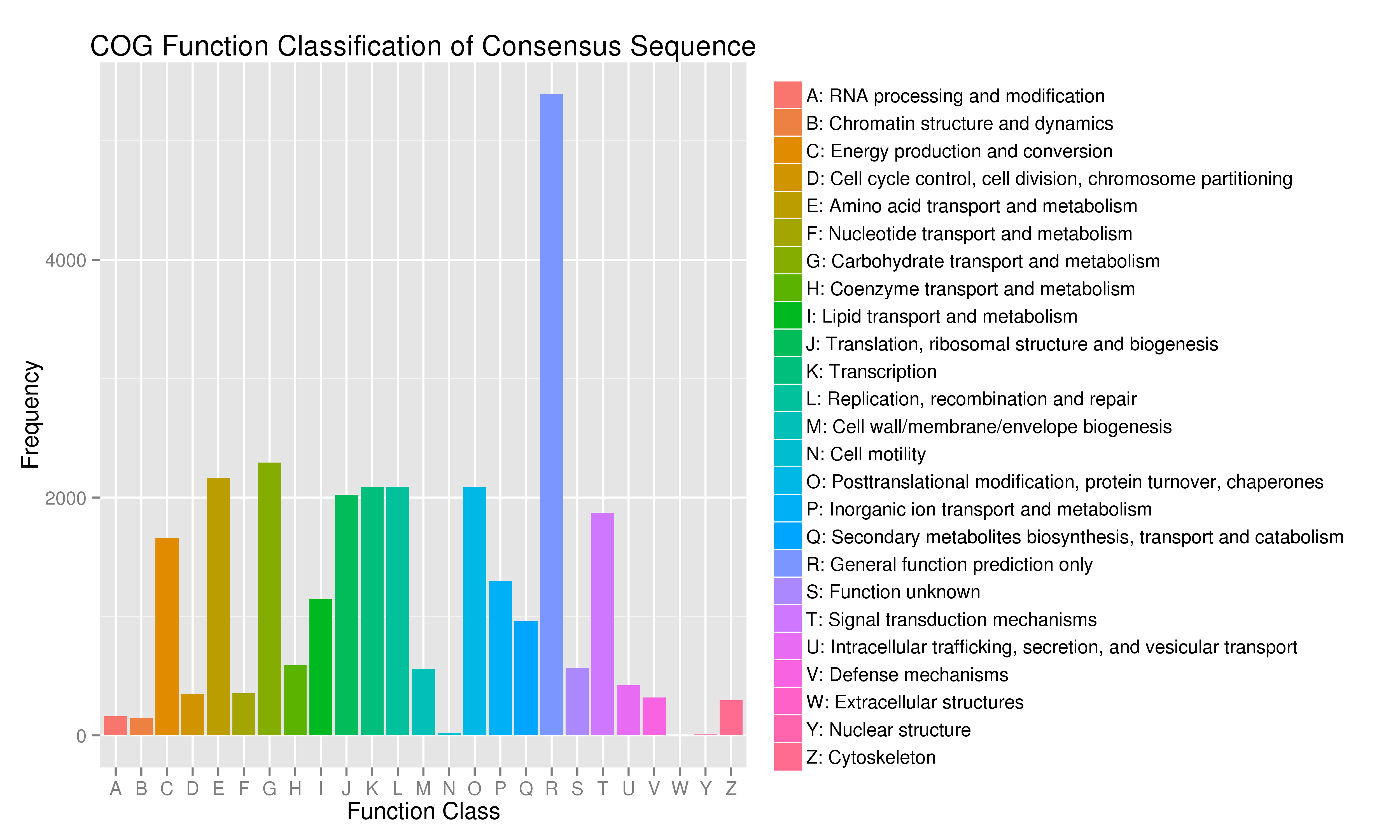


**Figure S1: COG classification statistics of *A. philoxeroides* annotated unigenes.**


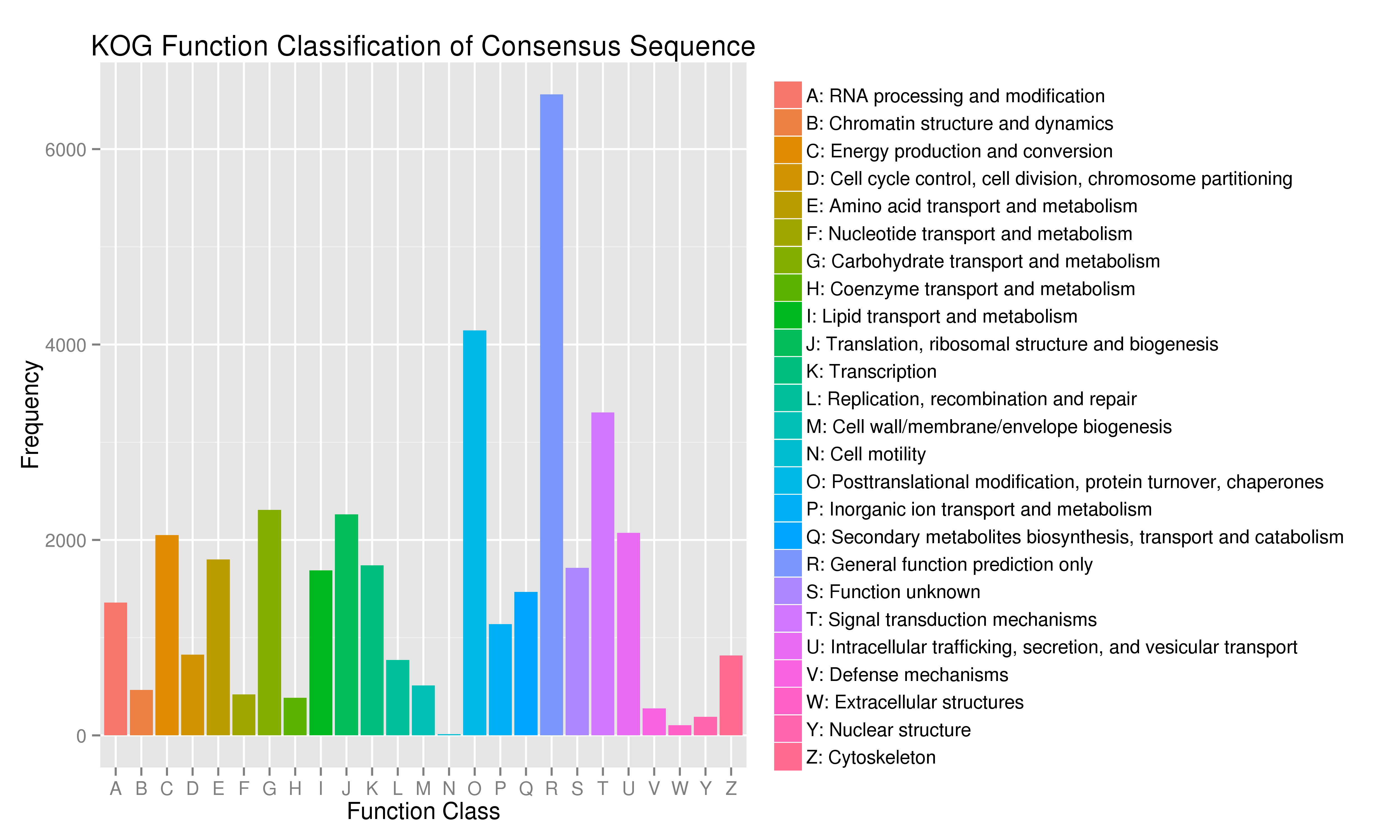


**Figure S2: KOG classification statistics of *A. philoxeroides* annotated unigenes.**





**Figure S3: Box plot of the *C*t values of four reference genes**Each box represents a set of treatments for an internal reference gene. The horizontal line in the box represents the median, the upper and lower quadrants of the box represent the upper/lower quartile, and the boxes at both ends represent the maximum and minimum values.

**Table S1 Construction of phylogenetic tree protein information**

| Protein name | Accession | Protein name | Accession |
| --- | --- | --- | --- |
| AtTPS13 | NP_193066.4 | CsKS1 | NP_001292675.1 |
| AtTPS21 | NP_001190374.1 | NtABS | NP_001312911.1 |
| SlTPS35 | NP_001307945.1 | PsiKS | ADB55710.1 |
| SlTPS32 | NP_001308023.1 | SlTPS24 | NP_001307929.1 |
| AtTPS10 | NP_179998.1 | SrKS | AAD34294.1 |
| SlTPS5 | NP_001233805.1 | CbLIS | Q96376.1 |
| SlTPS3 | NP_001295307.1 | VvNES/GLIS | NP_001268004.1 |
| SpPhS | NP_001310383.1 | AcNES1 | AER36088.1 |
| SoSAS | O81193.1 | AtLIS | AAO85533.1 |
| CmCPS1 | AAD04292.1 | AmNES/LIS2 | ABR24418.1 |
| PgCPS | ADB55707.1 | AmNES/LIS1 | ABR24417.1 |
| PsaKSA | O04408.1 | AmOCS | Q84NC8.1 |
| PsiCPS | ADB55709.1 | AmMYS1 | Q84ND0.1 |
| SlCPS | NP_001234008.2 | AmMYS2 | Q84NC9.1 |
| SrCPPS | AAB87091.1 | FaNES2 | P0CV95.1 |
| AgBIS | O81086.1 | FaNES1 | P0CV94.1 |
| AgLIMS | O22340.1 | SlTPS39 | NP_001306121.1 |
| AgMYS | O24474.1 | VvLIS/NES | AEY82696.1 |
| PaTPS-Far | AAS47697.1 | Vv3NES/GLIS | NP_001267990.1 |
| PaTPS-Lim | AAS47694.1 |  |  |

**Table S2 Quantitative RCR primers**

| **Primer name** | **Sequence from 5’ to 3’** | **Amplification product length** |
| --- | --- | --- |
| *ApTPS10*-54F20 | TGCTCAATCACCCCATTCCA | 168 bp |
| *ApTPS10*-202R20 | TGAAGAGCGCATAATCGTGG |  |
| *ApTPS14-*115F20 | TCACCACCTTCGCTCGATTT | 166 bp |
| *ApTPS14-* 261R20 | GGAGGGAAATGTTGCTACGC |  |
| *ApTPS15*-175F20 | AGCACTGGGGTCTATGTTGT | 133 bp |
| *ApTPS15*-288R20 | ACCCGCTGAAGTTACAATGC |  |
| *ApTPS16*-364F20 | GACTTTGACATGGCGCAGAT | 119 bp |
| *ApTPS16*-463R20 | TCCTCGCAAACACCAATTCC |  |
| *ApTPS12*-223F20 | TGTCCTCCAATGCAACCATG | 121 bp |
| *ApTPS12*-323R21 | AGCAATCCCCATCACAAAAGT |  |
| *ApTPS19*-1614F18 | AAGATACCACATTAGAGA | 213 bp |
| *ApTPS19*-1808R19 | CCATAACATAACGAAGAT |  |
| *UBC2*-F | AGCCCTGCTTTAACCATTTCC | 156 bp |
| *UBC2*-R | ATACTTCTGGGTCCAGCTCCTG |  |
| *ACT*-985F20 | TCACCAGAGTCCAGCACAAT | 261 bp |
| *ACT*-1226R20 | AGCAACTGGGATGACATGGA |  |
| *TUA*-980F20 | GGTGCTTTGAACGTGGATGT | 243 bp |
| *TUA*-1203R20 | CGGTACATGAGACAGCAAGC |  |
| *TUB*-536F20 | TTTGGGTGGAGGAACAGGTT | 175 bp |
| *TUB*-691R20 | TGAACAGAGAGGATGGCATT |  |

**Table S3 High homologous proteins with *A.philoxeroides* terpene synthase proteins**

| **Gene ID** | **Annotated function** | **Original name** | **E-value** | **Identity** | **Speices** | **Accession** |
| --- | --- | --- | --- | --- | --- | --- |
| *ApTPS1* | ent-kaurene synthase | C12696.graph_c0 | 4e^-82^ | 100% | *Oryza rufipogon* | ACD80291.1 |
| *ApTPS2* | ent-copalyl diphosphate synthase | C12706.graph_c0 | 3e^-62^ | 81% | *Ziziphus jujuba* | XP_015892068.1 |
| *ApTPS3* | sesquiterpene synthase | C1967.graph_c0 | 5e^-43^ | 41% | *Beta vulgaris subsp. vulgaris* | XP_010694277.1 |
| *ApTPS4* | sesquiterpene synthase | C22352.graph_c0 | 6e^-50^ | 49% | *Beta vulgaris subsp. vulgaris* | XP_010675322.1 |
| *ApTPS5* | (E,E)-alpha-farnesene synthase | C31092.graph_c0 | 0.0 | 100% | *Malus domestica* | NP_001280822.1 |
| *ApTPS6* | linalool synthase | C34291.graph_c0 | 4e^-177^ | 70% | *Xanthium strumarium* | AMP42990.1 |
| *ApTPS7* | Germacrene-D synthase | C38798.graph_c0 | 2e^-101^ | 49% | *Juglans regia* | XP_018822679.1 |
| *ApTPS8* | Valencene synthase | C52224.graph_c0 | 4e^-52^ | 59% | *Beta vulgaris subsp. vulgaris* | XP_010694308.1 |
| *ApTPS9* | germacrene A synthase | C54431.graph_c0 | 3e^-180^ | 49% | *Vitis vinifera* | ADR66821.1 |
| *ApTPS10* | (3S,6E)-nerolidol synthase | C57797.graph_c0 | 2e^-33^ | 44% | *Beta vulgaris subsp. vulgaris* | XP_010686917.1 |
| *ApTPS11* | ent-copalyl diphosphate synthase | C67779.graph_c0 | 3e^-56^ | 70% | *Nicotiana tabacum* | XP_016433900.1 |
| *ApTPS12* | (3S,6E)-nerolidol synthase | C714.graph_c0 | 2e^-51^ | 62% | *Beta vulgaris subsp. vulgaris* | XP_010665800.1 |
| *ApTPS13* | ent-copalyl diphosphate synthase | C73527.graph_c0 | 4e^-99^ | 58% | *Beta vulgaris subsp. vulgaris* | XP_010683963.1 |
| *ApTPS14* | (3S,6E)-nerolidol synthase | C73671.graph_c0 | 4e^-15^ | 50% | *Beta vulgaris subsp. vulgaris* | XP_010686920.1 |
| *ApTPS15* | (3S,6E)-nerolidol synthase | C76222.graph_c0 | 2e^-102^ | 73% | *Beta vulgaris subsp. vulgaris* | XP_010686920.1 |
| *ApTPS16* | (3S,6E)-nerolidol synthase | C77994.graph_c0 | 0.0 | 66% | *Beta vulgaris subsp. vulgaris* | XP_010686917.1 |
| *ApTPS17* | germacrene D synthase | C81677.graph_c0 | 6e^-174^ | 47% | *Beta vulgaris subsp. vulgaris* | XP_010675322.1 |
| *ApTPS18* | sesquiterpene synthase | C81757.graph_c0 | 0.0 | 82% | *Beta vulgaris subsp. vulgaris* | XP_010694277.1 |
| *ApTPS19* | (3S,6E)-nerolidol synthase | C82095.graph_c0 | 0.0 | 56% | *Beta vulgaris subsp. vulgaris* | XP_010686917.1 |
| *ApTPS20* | Ent-copalyl diphosphate synthase | C8570.graph_c0 | 2e^-94^ | 100% | *Oryza sativa Japonica* | BAD42452.1 |
| *ApTPS21* | sesquiterpene synthase | C85904.graph_c0 | 0.0 | 50% | *Santalum murrayanum* | F6M8H7.1 |
| *ApTPS22* | germacrene D synthase | C88870.graph_c2 | 0.0 | 71% | *Beta vulgaris subsp. vulgaris* | XP_010675322.1 |
| *ApTPS23* | Ent-kaurene synthase | C90613.graph_c0 | 0.0 | 71% | *Beta vulgaris subsp. vulgaris* | XP_010690598.1 |
| *ApTPS24* | Ent-kaurene synthase | C101822.graph_c0 | 2e^-101^ | 49% | *Juglans regia* | XP_018822679.1 |
| *ApTPS25* | Ent-copalyl diphosphate synthase | C104162.graph_c0 | 0.0 | 100% | *Oryza sativa Indica* | Q5MQ85.1 |
| *ApTPS26* | germacrene D synthase | C107950.graph_c0 | 7e^-55^ | 58% | *Citrus sinensis* | XP_006476905.1 |
| *ApTPS27* | Terpene synthase family, metal binding domain | C113666.graph_c0 | 3e^-26^ | 40% | *Mycena chlorophos* | GAT51557.1 |
| *ApTPS28* | Ent-copalyl diphosphate synthase | C114152.graph_c0 | 6e^-74^ | 100% | *Oryza sativa Japonica* | XP_015625954.1 |
| *ApTPS29* | sesquiterpene synthase | C122744.graph_c0 | 3e-44 | 51% | *Spinacia oleracea* | XP_021837075.1 |
| *ApTPS30* | Ent-copalyl diphosphate synthase | C129732.graph_c0 | 4e^-54^ | 78% | *Beta vulgaris subsp. vulgaris* | XP_010683963.1 |
| *ApTPS31* | beta-ocimene synthase | C143442.graph_c0 | 2e^-53^ | 49% | *Matricaria chamomilla var. recutita* | I6RE61.1 |
| *ApTPS32* | sesquiterpene synthase | C146968.graph_c0 | 7e^-43^ | 59% | *Beta vulgaris subsp. vulgaris* | XP_010678545.1 |
| *ApTPS33* | syn-CDP synthase | C150538.graph_c0 | 1e^-61^ | 100% | *Oryza sativa Japonica* | BAD42451.1 |
| *ApTPS34* | 7,15-Syn-pimaradiene synthase | C153619.graph_c0 | 8e^-87^ | 100% | *Oryza sativa Indica* | Q66QH3.1 |

**Table S4: the leaf areas consumed by different numbers of adults in 1 h**

| Adult number | Leaf areas consumed by adults (mm^2^) |
| --- | --- |
| 1 female+1 male | 1145.83±572.91 b |
| 2 female+2 male | 2027.04±506.76 ab |
| 3 female+3 male | 2604.93±434.15 a |

Note: The data in the table are presented as means ± SE. Different lowercase letters indicate a statistical difference between using different adult numbers, determined using a one-way ANOVA and a Tukey’s post-hoc test (*P* < 0.05) (*P* < 0.05).
